# Kaempferol Has Potent Protective and Antifibrillogenic Effects for α-Synuclein Neurotoxicity In Vitro

**DOI:** 10.3390/ijms222111484

**Published:** 2021-10-25

**Authors:** Masatoshi Inden, Ayaka Takagi, Hazuki Kitai, Taisei Ito, Hisaka Kurita, Ryo Honda, Yuji O. Kamatari, Sora Nozaki, Xiaopeng Wen, Masanori Hijioka, Yoshihisa Kitamura, Isao Hozumi

**Affiliations:** 1 Laboratory of Medical Therapeutics and Molecular Therapeutics, Gifu Pharmaceutical University, 1-25-4 Daigaku-nishi, Gifu 501-1196, Japan; 175066@gifu-pu.ac.jp (A.T.); 175044@gifu-pu.ac.jp (H.K.); 156004@gifu-pu.ac.jp (T.I.); kurita@gifu-pu.ac.jp (H.K.); hozumi@gifu-pu.ac.jp (I.H.); 2 The United Graduate School of Drug Discovery and Medical Information Sciences, Gifu University, 1-1 Yanagido, Gifu 501-1193, Japan; ryohonda.rh@gmail.com (R.H.); kamatari@gifu-u.ac.jp (Y.O.K.); 3 Institute for Glyco-core Research (iGCORE), Gifu University, 1-1 Yanagido, Gifu 501-1193, Japan; 4 Life Science Research Center, Gifu University, 1-1 Yanagido, Gifu 501-1193, Japan; 5 Laboratory of Pharmacology and Neurobiology, College of Pharmaceutical Sciences, Ritsumeikan University, 1-1-1 Noji-Higashi, Kusatsu 525-8577, Japan; ph0106hh@ed.ritsumei.ac.jp (S.N.); xiaopeng@fc.ritsumei.ac.jp (X.W.); mhijioka@med.nagoya-cu.ac.jp (M.H.); yo-kita@fc.ritsumei.ac.jp (Y.K.); 6 Department of Neurocognitive Science, Institute of Brain Science, Nagoya City University Graduate School of Medical Sciences, 1 Kawasumi, Mizuho-cho, Mizuho-ku, Nagoya 467-8601, Japan

**Keywords:** α-synuclein, synucleinopathies, kaempferol, Parkinson’s disease, dementia with Lewy bodies, multiple system atrophy, autophagy

## Abstract

Aggregation of α-synuclein (α-Syn) is implicated in the pathogenesis of Parkinson’s disease (PD), dementia with Lewy bodies (DLB), and multiple system atrophy (MSA). Therefore, the removal of α-Syn aggregation could lead to the development of many new therapeutic agents for neurodegenerative diseases. In the present study, we succeeded in generating a new α-Syn stably expressing cell line using a piggyBac transposon system to investigate the neuroprotective effect of the flavonoid kaempferol on α-Syn toxicity. We found that kaempferol provided significant protection against α-Syn-related neurotoxicity. Furthermore, kaempferol induced autophagy through an increase in the biogenesis of lysosomes by inducing the expression of transcription factor EB and reducing the accumulation of α-Syn; thus, kaempferol prevented neuronal cell death. Moreover, kaempferol directly blocked the amyloid fibril formation of α-Syn. These results support the therapeutic potential of kaempferol in diseases such as synucleinopathies that are characterized by α-Syn aggregates.

## 1. Introduction

α-Synuclein (α-Syn) is a small 14 kDa protein encoded by the *SNCA* gene. Parkinson’s disease (PD), dementia with Lewy bodies (DLB), and multiple system atrophy (MSA) represent the synucleinopathies, an umbrella term for the group of disorders related to the aggregation of α-Syn [1]. The misfolding and aggregation of α-Syn in neurons, neuronal processes, or glial cells are thought to be critical pathogenic events in synucleinopathies. In PD and DLB, α-Syn inclusions are detected as Lewy bodies and Lewy neurites in subcortical and cortical neurons; in MSA, α-Syn inclusions are mostly distributed in glial cells and are referred to as glial cytoplasmic inclusions [1,2,3]. However, the functions of α-Syn have yet to be fully and accurately clarified. α-Syn, which is located in synaptic terminals and neuronal nuclei in both the central and peripheral nervous system, is typically viewed as a neuronal protein involved in neurotransmission. Under healthy conditions, α-Syn is a naturally unfolded and soluble monomer, although its presence as a tetramer has previously been discussed [4,5]. However, during the pathological process, the structural change of α-Syn from an α-helix to a β-sheet leads to oligomer, protofibril, and mature fibril formation. Oligomers or protofibrils of α-Syn are regarded as toxic species that induce neuronal cell death. In addition, point mutations and gene multiplications of *SNCA* modify the aggregation potential of α-Syn [6,7]. These mutations include amino acid substitutions, such as A53T, A30P, and E46K, as well as gene duplication and triplication events that cause autosomal-dominant PD in a dose-dependent manner; thus, α-Syn is clearly correlated with the disease process [5]. Moreover, variants of *SNCA* that lead to α-Syn expression show a genetic risk factor for sporadic PD.

Kaempferol is a flavonoid found in many fruits, vegetables, and medicinal plants. It has numerous biological and pharmacological properties, such as anti-inflammatory, antioxidative, and neuroprotective properties against various diseases including neurodegenerative diseases such as PD [8,9]. PD models can be divided into those that use environmental or synthetic neurotoxins and those that use in vivo expression of PD-related mutations discovered in human patients. Generally, neurotoxins such as rotenone, 6-hydroxydopamine, and 1-methyl-4-phenyl-1,2,3,6-tetrahydropyridine (MPTP) are used to create PD models with rodents and nonhuman primates. In MPTP-treated mice, kaempferol was shown to exert antioxidative activity that led to improved motor function and increased levels of striatal dopamine (DA) as well as its metabolites [10]. Kaempferol was also reported to increase the resistance of DA neurons to neuroinflammation [11]. In addition, kaempferol apparently prevented lipid droplet toxicity in vitro and ameliorated DA neuronal loss and behavioral deficits in a PD mouse model; these effects were found to be autophagy-dependent [11,12]. However, the effect of kaempferol on α-Syn-related neurotoxicity induced via the autophagy pathway has not yet been investigated.

Our previous study showed that kaempferol contributes to the clearance of aggregations of mutant copper–zinc superoxide dismutase 1 proteins (SOD1^mut^; the encoding gene of which is causative for amyotrophic lateral sclerosis) via the activation of the autophagic pathway; moreover, we found that kaempferol has a neuroprotective effect against SOD1^mut^-induced neurotoxicity [13]. Therefore, we hypothesized that kaempferol inhibits α-Syn-related neurotoxicity and prevents DA neurodegeneration via the activation of the autophagic pathway. In the present study, we examined whether kaempferol provided significant protection against α-Syn-related neurotoxicity by inhibiting α-Syn accumulation. We also investigated whether kaempferol induced autophagy through an increase in the biogenesis of lysosomes by inducing the expression of transcription factor EB (TFEB). Finally, we assessed whether kaempferol directly inhibits the formation of amyloid fibrils.

## 2. Results

### 2.1. Establishment of a Cellular Model with Cumate-Induced α-Syn Expression

**Figure 1:**
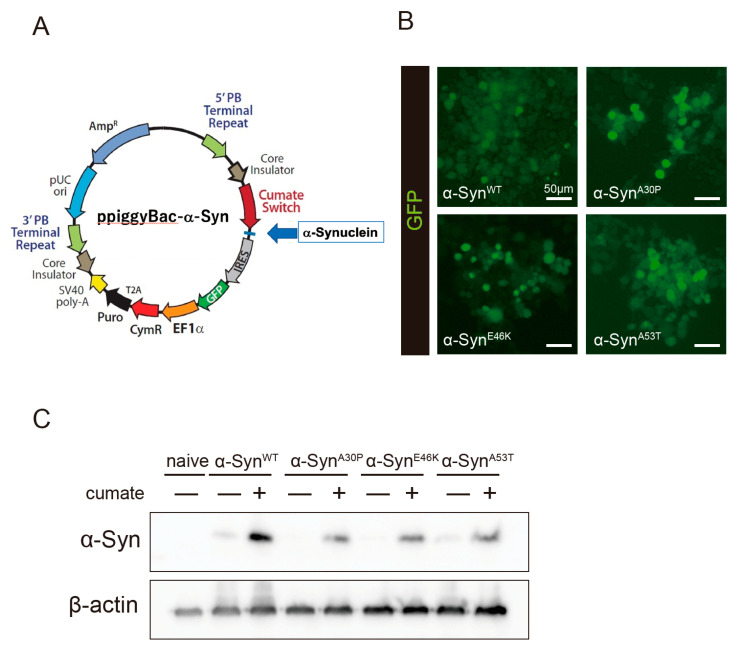
Establishment of a cumate-induced α-Syn expression system. (**A**) PiggyBac cumate-α-Syn transgene vector. (**B**) Confocal imaging of GFP in α-Syn-N2a cells, which were cotransfected with the piggyBac cumate-α-Syn transgene vector and piggyBac transposase construct after exposure to 50 µg/mL of cumate for 48 h. Scale bar: 50 µm. (**C**) Immunoblot analysis of α-Syn protein levels after exposure to 50 µg/mL of cumate for 48 h.

To evaluate the effect of kaempferol against α-Syn-related neurotoxicity, a piggyBac transposon system was used to stably express α-Syn in N2a cells in a cumate-inducible manner. After cotransfection of the piggyBac cumate-α-Syn transgene vector (Figure 1A) and piggyBac transposase construct into N2a cells, stable cells were selected using puromycin (hereafter, named α-Syn-N2a cells). In the presence of cumate, GFP expression was induced in the α-Syn-N2a cells (Figure 1B). The GFP-positive cells were then sorted using a cell sorter to obtain α-Syn-N2a cells of high purity. In these cells, the expressions of wild-type α-Syn (α-Syn^WT^) or mutant-type α-Syn (α-Syn^mut^), including α-Syn^A53T^, α-Syn^A30P^, and α-Syn^E46K^, were significantly upregulated after exposure to cumate for 48 h (Figure 1C). We experienced difficulty in detecting higher-molecular-weight species of α-Syn or phospho-S129 α-Syn in our experimental system (data not shown).

### 2.2. Kaempferol Provided Protection against α-Syn-Associated Neurotoxicity

**Figure 2:**
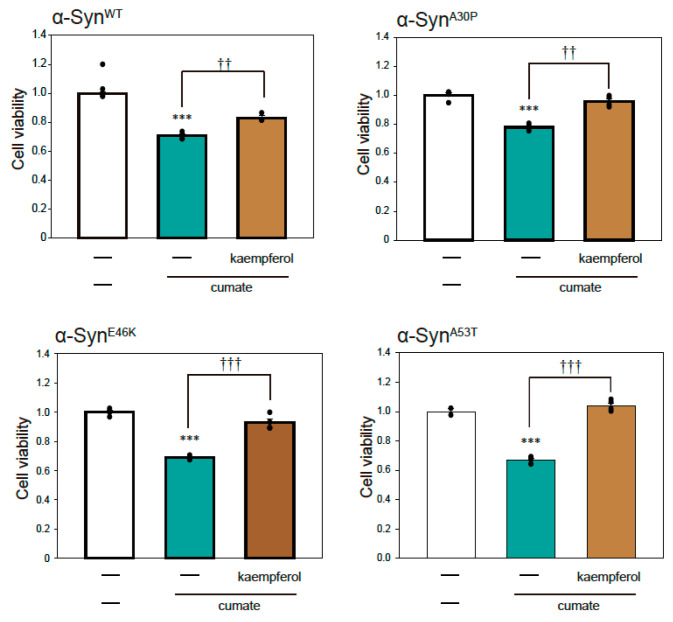
Effect of kaempferol against α-Syn-associated neurotoxicity. α-Syn-N2a cells were treated with 5 µM kaempferol in the presence or absence of 50 µg/mL of cumate for 48 h. Cell viability was measured using an MTT assay. Data are expressed as means ± SEM from three independent experiments. *** *p* < 0.001 vs. α-Syn-N2a cells in the absence of 50 µg/mL cumate; †† *p* < 0.01 and ††† *p* < 0.001 vs. α-Syn-N2a cells in the presence of 50 µg/mL cumate.

To examine α-Syn-mediated neurotoxicity in α-Syn-N2a cells after exposure to cumate, we performed a thiazolyl blue tetrazolium bromide (MTT) assay. As shown in Figure 2, cell death was induced by the induction of α-Syn^mut^ expression as well as α-Syn^WT^ expression by cumate treatment. These results suggest that we established a PD model cell in which cell death is triggered by the expressions of α-Syn^WT^ and α-Syn^mut^.

To investigate the effect of kaempferol against α-Syn-related neurotoxicity, we performed an MTT assay (Figure 2). Kaempferol significantly inhibited α-Syn^WT^-induced neurotoxicity (Figure 2). Similarly, kaempferol prevented α-Syn^mut^-related neuronal cell death. Thus, kaempferol appears to have significant neuroprotective effects against α-Syn-induced neurotoxicity.

### 2.3. Effect of Kaempferol on α-Syn via Activation of Autophagy

**Figure 3:**
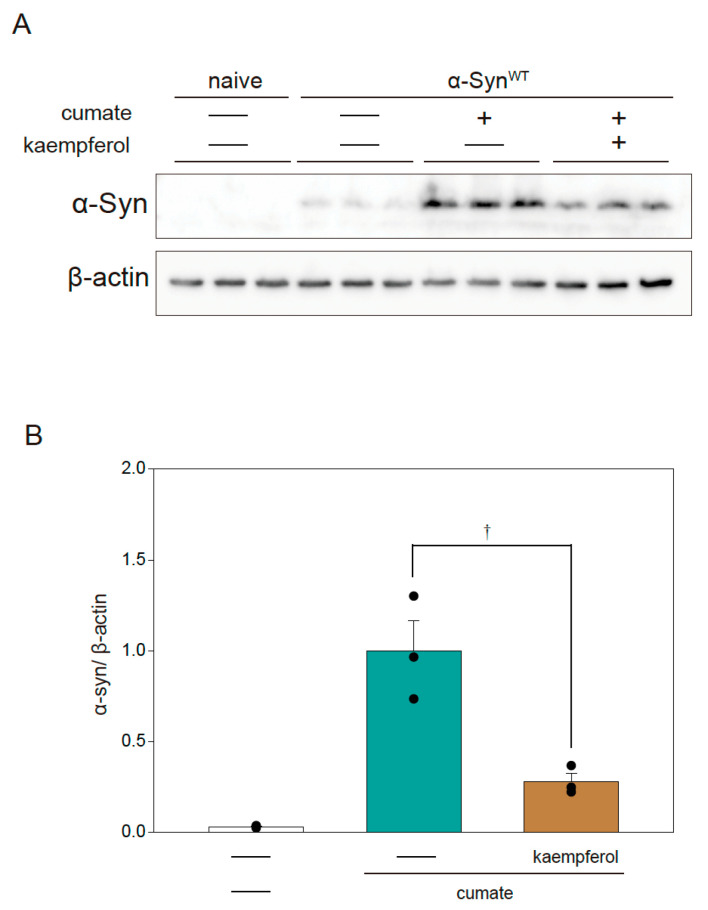
Effect of kaempferol on α-Syn protein levels in α-Syn^WT^-N2a cells. α-Syn^WT^-N2a cells were treated with 5 µM kaempferol in the presence or absence of 50 µg/mL of cumate for 48 h. Immunoblotting was then performed. (**A**) Immunoblot analysis of α-Syn. (**B**) Densitometric quantification of α-Syn. Data are expressed as means ± SEM from three independent experiments. † *p* < 0.05 vs. α-Syn^WT^-N2a cells in the presence of 50 µg/mL cumate.

The autophagy pathway is closely involved in the degradation of α-Syn protein [14]. Our previous study revealed that kaempferol induces autophagy [13]. To examine the relationship between the protective effect of kaempferol and the expression of α-Syn protein, Western blot analysis was performed in α-Syn^WT^-N2a cells (Figure 3A,B). Kaempferol remarkably reduced α-Syn protein levels in α-Syn^WT^-N2a cells after exposure to cumate for 48 h.

**Figure 4:**
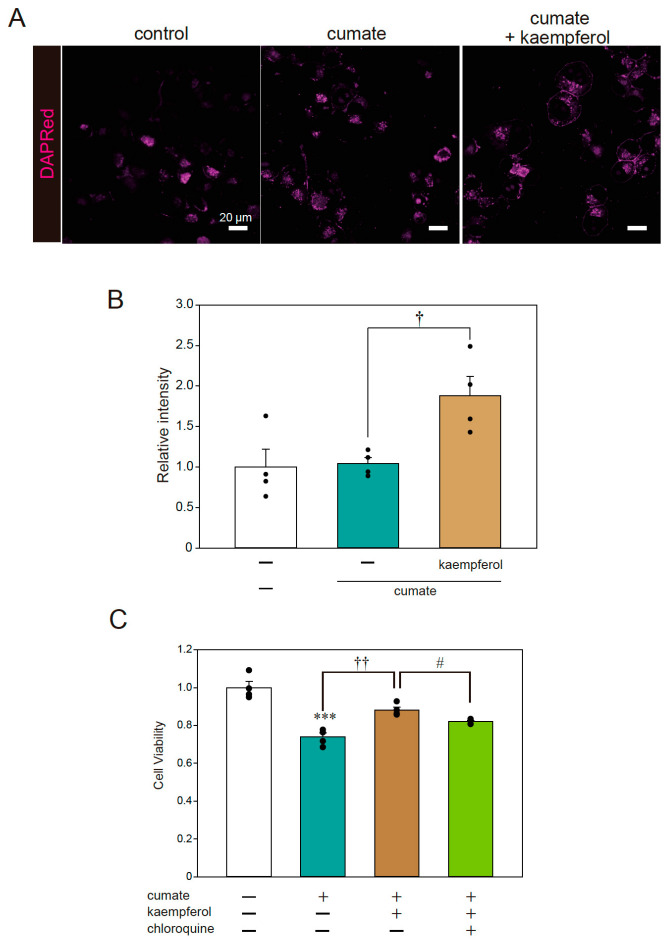
Kaempferol activated autophagy. α-Syn^WT^-N2a cells were treated with 5 µM kaempferol in the presence or absence of 50 µg/mL of cumate for 48 h. Fluorescence imaging was then performed with DAPRed staining. (**A**) Imaging of DAPRed. Scale bar: 20 µm. (**B**) Quantified analysis of imaging. (**C**) α-Syn^WT^-N2a cells were treated with 5 µM kaempferol in the presence or absence of 50 µg/mL of cumate for 48 h and with chloroquine (200 nM). Cell viability was determined by an MTT assay. Data are expressed as means ± SEM from four independent experiments. *** *p* < 0.001 vs. α-Syn^WT^-N2a cells in the absence of 50 µg/mL cumate; † *p* < 0.05 and †† *p* < 0.01. vs. α-Syn^WT^-N2a cells in the presence of 50 µg/mL cumate. # *p* < 0.05 vs. α-Syn^WT^-N2a cells in the presence of 50 µg/mL cumate with kaempferol.

Fluorescence imaging was also performed to examine the signal of DAPRed, a selective marker of autophagosomes and autolysosomes. The representative photomicrographs in Figure 4A,B show that the DAPRed signal was significantly increased with kaempferol treatment in α-Syn^WT^-N2a cells; thus, kaempferol appeared to induce autophagy, consistent with the results of our previous study [13]. To further investigate whether autophagy is involved in the neuroprotective effects of kaempferol against α-Syn-related neurotoxicity, an MTT assay was performed using chloroquine as an autophagy inhibitor. The protective effect of kaempferol was significantly abolished by chloroquine treatment (Figure 4C). These results suggest that kaempferol reduces intracellular α-Syn protein levels via the activation of autophagy and subsequently suppresses α-Syn-induced neurotoxicity.

### 2.4. Effect of Kaempferol on Lysosomal Activation via TFEB

**Figure 5:**
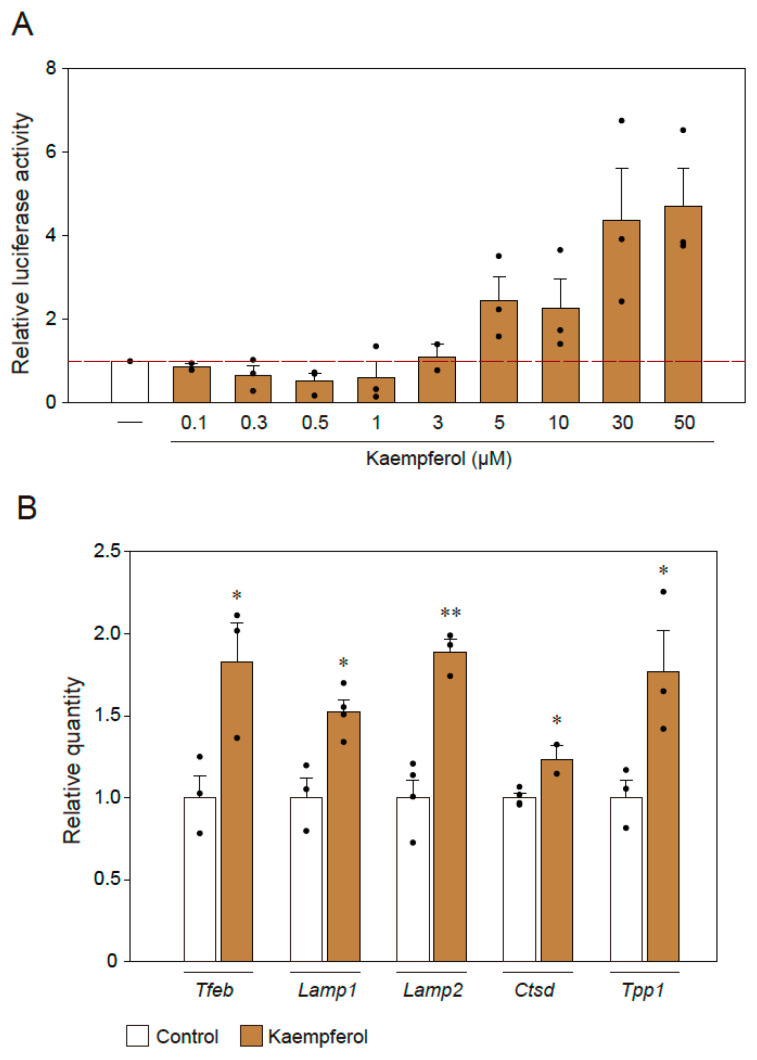
TFEB is involved in the kaempferol-induced activation of autophagy. (**A**) Results of 4 × CLEAR luciferase assays. (**B**) qRT-PCR analysis of the effects of kaempferol on the mRNA expression levels of *Tfeb*, *Lamp1*, *Lamp2*, *Ctsd*, and *Tpp1*. Data are expressed as means ± SEM from three independent experiments. * *p* < 0.05 and ** *p* < 0.01 vs. the control.

According to our previous study, kaempferol induces autophagy via AMP-activated protein kinase (AMPK), i.e., the mammalian target of the rapamycin (mTOR) pathway [13]. To identify the downstream signaling of the mTOR–AMPK pathway mediated by kaempferol, we investigated TFEB, which is a transcription factor that is a well-known master regulator of autophagy and lysosomal biogenesis processes [15]. First, 4 × CLEAR luciferase assays were used to representatively assess the activity of TFEB-regulated genes [16]. In this assay, the binding of TFEB to the 4 × repeated CLEAR-response element promotes the expression of luciferase, the levels of which are determined in dual-luciferase reporter assays. We found that CLEAR reporter expression was significantly activated by kaempferol treatment in a concentration-dependent manner (Figure 5A). In addition, we used qRT-PCR to analyze the mRNA levels of autophagy-associated genes for which transcription could be regulated by TFEB (Figure 5B). The mRNA levels of *Tfeb*, *Lamp1*, *Lamp2*, *Ctsd*, and *Tpp1* were significantly upregulated in response to kaempferol treatment (Figure 5B).

**Figure 6:**
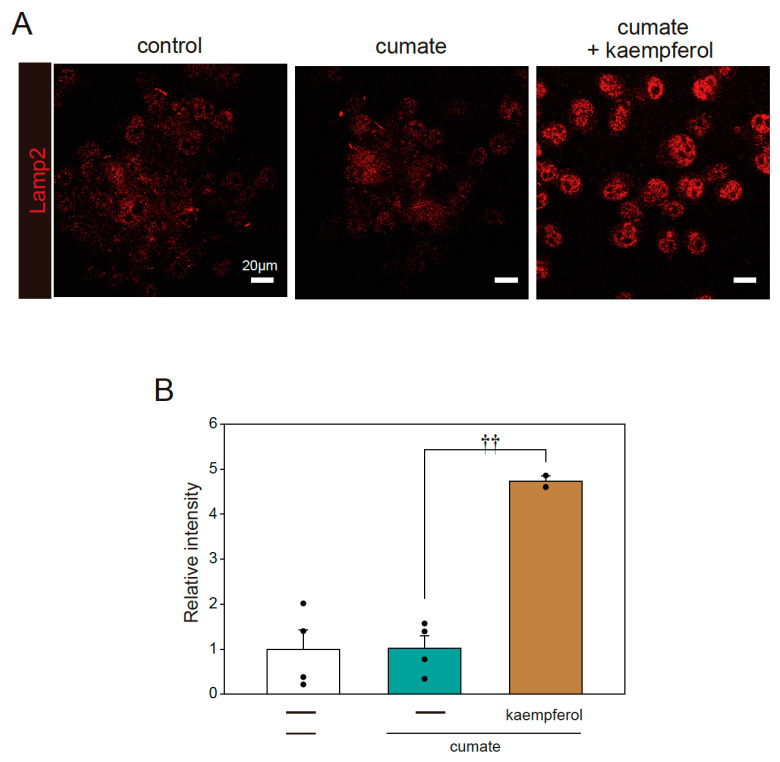
Kaempferol enhances lysosomal function. α-Syn^WT^-N2a cells were treated with 5 µM kaempferol in the presence or absence of 50 µg/mL of cumate for 48 h. Immunofluorescence staining with Lamp2 antibody was then performed. (A) Imaging of Lamp2. Scale bar: 20 µm. (B) Quantified analysis of imaging. Data are expressed as means ± SEM from four independent experiments. †† *p* < 0.01 vs. α-Syn^WT^-N2a cells in the presence of 50 µg/mL cumate.

In further analysis, we determined whether kaempferol increases lysosomal biogenesis using immunofluorescence staining with Lamp2 antibody. Kaempferol treatment accelerated lysosomal activation, as shown by the quantification of fluorescence intensity in Lamp2-stained cells (Figure 6A,B). These results indicate that kaempferol promotes the function of autophagy via the nuclear transposition of TFEB.

### 2.5. Effect of Kaempferol on the Amyloid Fibril Formation of α-Syn

**Figure 7:**
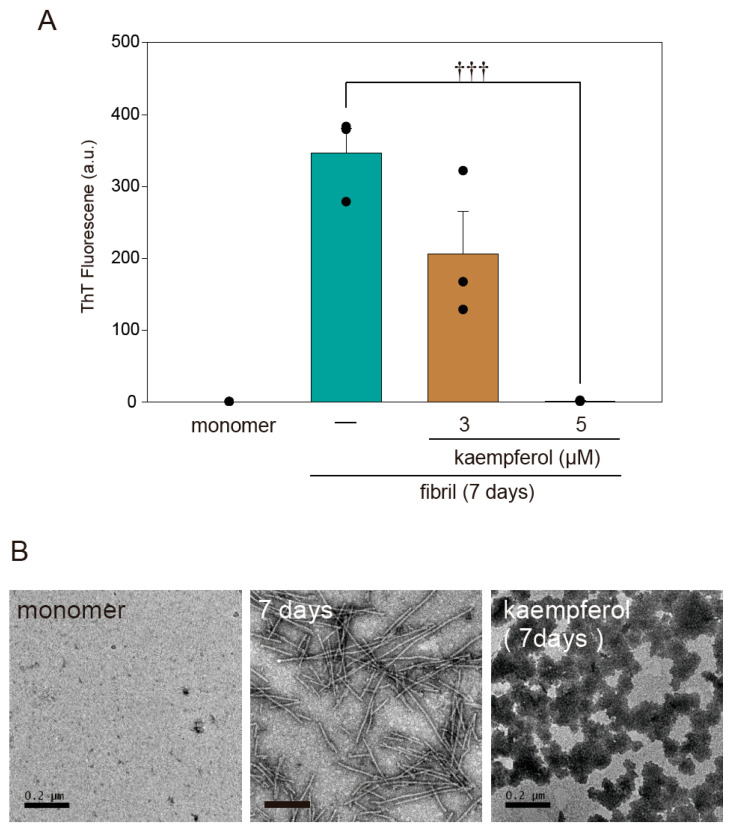
Kaempferol inhibits amyloid fibril formation of α-Syn. (**A**) Results of a thioflavin T assay. Amyloid fibril formation of α-Syn in the presence or absence of kaempferol. (**B**) Representative TEM images. Scale bar: 0.2 µm. Data are expressed as means ± SEM from three independent experiments. ††† *p* < 0.001 vs. recombinant α-Syn in the absence of kaempferol.

A previous study found that flavonoids prevent the onset of amyloid fibril formation of α-Syn [17]. To investigate whether kaempferol directly inhibits amyloid fibril formation, we performed a thioflavin T assay as well as transmission electron microscopy (TEM) imaging using recombinant α-Syn^WT^ protein. According to both thioflavin T fluorescence (Figure 7A) and TEM (Figure 7B), kaempferol inhibited the amyloid fibril formation of α-Syn. In addition, kaempferol induced the formation of a structure resembling an amorphous aggregation that had low cytotoxicity [14]. These results suggest that kaempferol directly inhibits the amyloid fibril formation of α-Syn.

## 3. Discussion

The goal of the present study was to investigate whether kaempferol inhibits α-Syn-related neurotoxicity and prevents DA neurodegeneration via the activation of the autophagic pathway. We demonstrated that kaempferol provided significant protection against α-Syn-related neurotoxicity by inhibiting α-Syn accumulation. We also showed that kaempferol induced autophagy through an increase in the biogenesis of lysosomes by inducing TFEB expression and reducing the accumulation of α-Syn; thus, neuronal cell death was prevented. Moreover, kaempferol directly blocked the amyloid fibril formation of α-Syn. Collectively, these results support the therapeutic potential of kaempferol in the treatment of diseases that are characterized by α-Syn aggregates such synucleinopathies including PD.

The mechanism of the neuroprotective effects of flavonoids, including kaempferol, is associated with the modulation of autophagy [18]. Autophagy is a multistep process involving induction, phagophore formation, sequestration, autophagosome formation, and finally fusion of the autophagosome with a lysosome to form an autophagolysosome, which then induces either the death or survival pathway [19]. Flavonoids can trigger autophagy via various mechanisms through the canonical (Beclin-1-dependent) and noncanonical (Beclin-1-independent) routes of autophagy, although these flavonoid-activated mechanisms are yet to be clarified. Previously, we reported that kaempferol clearly induced autophagy via the AMPK–mTOR pathway [13]. In the present study, we found that kaempferol promote the function of autophagy via the nuclear transposition of TFEB, a downstream signal of the AMPK–mTOR pathway. Interestingly, kaempferide, another flavonoid, did not induce autophagy via the AMPK–mTOR pathway [13]. The difference between the chemical structures of kaempferol and kaempferide is the functional group in the B ring; kaempferol and kaempferide are framed with the B ring 4′-OH group and 4′-methoxy group, respectively. Therefore, the 4′-OH group in the B ring of kaempferol may be related to the induction of autophagy [13].

As previously discussed, kaempferol has several biological and pharmacological properties that function against many diseases including neurodegenerative diseases [8,9,13]. Among them, the antioxidant effect of kaempferol is widely known [8,9,13]. In our previous paper, we showed that kaempferol directly removes hydroxyl radicals and superoxide anions [13]. It was reported that α-Syn aggregates can induce oxidative stress in mitochondria [20]. In the present experiment, not only the activation of autophagy via TFEB but also the ability of antioxidation of kaempferol seem to be important factors for producing its neuroprotective effect.

In previous studies [10,11,12], kaempferol has shown neuroprotective effects in animal models of PD. In these studies, the researchers used a concentration of 5 µM or higher of kaempferol. Kaempferol and other flavonoids, which are similar in structure to kaempferol, also showed neuroprotective effects in in vivo experimental models of CNS diseases [9]. These results suggest that kaempferol passes through the BBB to produce its effects.

The onset of amyloid fibril formation of α-Syn is a key pathological event in synucleinopathies. Recent studies on α-Syn identified the existence of different strains that exhibit distinct properties such as differences in the structure, toxicity, and ability to seed and propagate [14]. Differences in α-Syn strains also exist between synucleinopathies. In addition, oligomers and fibrils of 

α-Syn are thought to be formations that are more neurotoxic, and they are presumed to spread throughout the brain of patients with PD along neuroanatomical connections [14]. Therefore, a variety of therapeutic modalities have been conceived and developed to target α-Syn protein levels and aggregate formation. In the present study, kaempferol substantially reduced the accumulation of α-Syn via the activation of autophagy. The results of the thioflavin T fluorescence assays also demonstrated that kaempferol reduced the β-sheet content of amyloid fibril formation of α-Syn. Moreover, the TEM results confirmed that the amyloid fibril formation of α-Syn was altered by kaempferol to form a structure resembling an amorphous aggregation that had low cytotoxicity [14,21] or made it more easily degradable by autophagy. Thus, the present results indicate that kaempferol is a candidate prototype for the development of drugs that could prevent the aggregation of amyloid fibril formation of α-Syn in synucleinopathies.

The current view of PD comprises the concept that α-Syn aggregates can trans-synaptically spread from neuron to neuron in a prion-like fashion [22,23]. α-Syn aggregates can propagate from the peripheral nervous system to the brain via the enteric nervous system [24] or the sensory nervous system [23,25]. The release of α-Syn, as with other cytosolic proteins, does not occur via the endoplasmic reticulum (ER)-Golgi biosynthetic/secretory pathway, but may be, at least in part, mediated by exosomes or other extracellular vesicles (EVs) [26]. In addition, the autophagy–lysosome pathway inhibition increases the ratio of extra- to intracellular α-Syn and upregulates SNCA association with EVs in neuronal cells, which might offer multiple therapeutic targets [26]. Thus, we speculate kaempferol could potentially modulate the pathological transneuronal spread of α-Syn via the activation of lysosomal biogenesis.

In conclusion, kaempferol was found to have protective effects against α-Syn-related neurotoxicity by enhancing lysosomal function and activation of autophagy via TFEB. Aggregation of α-Syn is closely related not only to the development of motor diseases such as PD, but also to the development of dementia such as DLB. Therefore, since kaempferol is contained in food and can be easily consumed before the onset of these diseases, it may contribute in terms of prevention and is promising as a one-seed compound.

## 4. Materials and Methods

### 4.1. Plasmid, Cell Culture, and Stable Cells

Wild-type human α-Syn cDNA was purchased from TransOMIC Technologies (Huntsville, AL, USA), and subcloned into the piggyBac cumate switch inducible vector (PBQM812A-1, System Biosciences, Palo Alto, CA, USA) between the NheI/NotI sites (ppiggyBac-α-Syn^WT^). The mutant α-Syn (A53T, A30P, and E46K) genes were generated by Quick Change site-directed mutagenesis (Stratagene, La Jolla, CA, USA) according to the manufacturer’s protocol. The primer pairs were as follows: 5′-GGTGTGACAACAGTGGCTGAGAAGACC-3′ and 5′-GCAGGATCCGGTTGGGCGATCCCAATTACACC-3′ for ppiggyBac-α-Syn^A53T^, 5′-GAAGCACCAGGAAAGACAAAAGAGGGT-3′ and 5′-CTTTCCTGGTGCTTCTGCCACACCCTG-3′ for ppiggyBac-α-Syn^A30P^, and 5′-ACCAAGAAGGGAGTGGTGCATGGTGTG-3′ and 5′-CACTCCCTTCTTGGTTTTGGAGCCTAC-3′ for ppiggyBac-α-Syn^E46K^. To establish the transgene-expressing cell lines (α-Syn-N2a cells), each α-Syn expression vector was cotransfected with the piggyBac transposase into N2a cells according to the manufacturer’s protocol (System Biosciences). After cotransfection, stable cells were selected using puromycin. In the presence of cumate, GFP-positive cells were sorted using a cell sorter (SH800, SONY, Tokyo, Japan).

N2a cells (Mouse Albino neuroblastoma; ECACC, UK) were maintained in Dulbecco’s modified eagle medium (Wako Pure Chemical Industries Ltd., Tokyo, Japan) containing 10% (v/v) fetal bovine serum (Thermo Fisher Inc., Waltham, MA, USA) under a humidified atmosphere of 5% CO_2_ at 37 °C. The cells were passaged by trypsinization every 3–4 days.

### 4.2. MTT Assay

The number of live cells was estimated using a Cell Counting Kit-8 following the manufacturer’s protocol (Wako Pure Chemical Industries Ltd.) as previously described [13,15]. α-Syn-N2a cells were treated with 5 µM kaempferol (Wako Pure Chemical Industries) in the presence or absence of 50 µg/mL of cumate for 48 h, and cell viability was then measured using a Cell Counting Kit-8 (CCK8). CCK8 reagent was added into the wells and the plate was incubated at 37 °C for 4 h. The optical density of formazan was detected at 450 nm by GloMax^®^ (Promega, Madison, WI, USA) for calculating cell viability. The wavelength of 600 nm was used as the reference.

### 4.3. Immunoblotting

Biochemical analysis was performed as previously described [13,15]. For immunoblotting, rabbit monoclonal anti-alpha-synuclein (1:5000) was purchased from Abcam (Cambridge Biomedical Campus, Cambridge, UK), rabbit polyclonal anti-LC-3 (1:1000) was purchased from Cell Signaling Technology (Danvers, MA, USA), and mouse monoclonal anti-β-actin (1:5000) was purchased from Sigma-Aldrich (St. Louis, MO, USA). After the primary antibody reaction, the membrane was then incubated with the following secondary antibodies at RT for 30 min: goat anti-rabbit IgG antibody, peroxidase conjugated, H+L (Merck KGaA, Darmstadt, Germany) (1:2000 dilution), or goat anti-mouse IgG antibody, peroxidase conjugated, H+L (Merck KGaA) (1:2000 dilution). Finally, the membrane was incubated with ECL prime (GE Healthcare) to generate chemiluminescence. Chemiluminescence was detected by using a Fusion System (Vilber-Lourmat, Marne-la-Vallée, France). The band density was measured by ImageJ software (NIH, New York, NY, USA).

### 4.4. Fluorescence Imaging

Fluorescence imaging was performed using DAPRed according to the manufacturer’s protocol (Dojindo Molecular Technologies Inc., Kumamoto, Japan). Immunofluorescence staining was performed with anti-Lamp2 antibody (1:500; Gene Tex, Alton Pkwy Irvine, CA, USA) based on the method from a previous study [Ito et al., 2020]. Fluorescent microscopy images were acquired using a confocal fluorescence microscope (LSM700, Carl Zeiss, Jena, Germany) and using ImageJ for image analysis, the fluorescence intensities in each image were calculated.

### 4.5. RNA Preparation and qRT-PCR

RNA preparation and qRT-PCR were performed as previously described [15]. Reverse transcription was performed using the ReverTra Ace qPCR RT Master Mix, in accordance with the manufacturer’s instructions (TOYOBO, Osaka, Japan). qRT-PCR was performed using SYBR Green on a StepOne Real-Time PCR System, in accordance with the manufacturer’s instructions (Life Technologies, Carlsbad, CA, USA). The sequences of gene-specific primer sets were as follows: 5′cagcaggtggtgaagcaagagt3′ and 5′tccaggtgatggaacggagact3′ for *Tfeb*, 5′tcaaggtggacagtgacaggt3′ and 5′tgactcctcttcctgccaatga3′ for *Lamp1*, 5′tctccggttaaaggcgcaaag3′ and 5′tcatctcccattctgcataaaggc3′ for *Lamp2*, 5′cagatgccagaatcggaaggg3′ and 5′ggactcaatcagccggggat3′ for *Ctsd*, 5′cccatgttataaggtccccacatcc3′ and 5′ccaagtgcaggctaacagttcc3′ for *Tpp1*, and 5′cgttgacatccgtaaagacc3′ and 5′gctaggagccagagcagtaa3′ for β-actin. The expression levels of mRNA were normalized to the expression levels of β-actin mRNA.

### 4.6. Thioflavin T Assay and TEM Imaging

The thioflavin T assay and TEM imaging of the recombinant α-Syn protein were performed based on methods in a previous study [27].

### 4.7. Statistical Analysis

Data are presented as means ± standard error of the mean (SEM). Significance was determined using ANOVA. In post hoc comparisons, the Bonferroni or Dunn test was used to determine significance (SigmaPlot 13, Systat Software). *p*-values < 0.05 were considered statistically significant.
